# Epigenetic Matters: The Link between Early Nutrition, Microbiome, and Long-term Health Development

**DOI:** 10.3389/fped.2017.00178

**Published:** 2017-08-22

**Authors:** Flavia Indrio, Silvia Martini, Ruggiero Francavilla, Luigi Corvaglia, Fernanda Cristofori, Salvatore Andrea Mastrolia, Josef Neu, Samuli Rautava, Giovanna Russo Spena, Francesco Raimondi, Giuseppe Loverro

**Affiliations:** ^1^Department of Pediatrics, Aldo Moro University, Bari, Italy; ^2^Neonatology and Neonatal Intensive Care Unit, St. Orsola-Malpighi Hospital, Department of Medical and Surgical Sciences, University of Bologna, Bologna, Italy; ^3^Department of Biomedical Science and Human Oncology, Section of Obstetrics and Gynecology, Aldo Moro University, Bari, Italy; ^4^Division of Neonatology, Department of Pediatrics, University of Florida, Gainesville, FL, United States; ^5^Department of Pediatrics, University of Turku, Turku University Hospital, Turku, Finland; ^6^Division of Neonatology, Department of Translational Medical Sciences, University “Federico II” di Napoli, Naples, Italy

**Keywords:** epigenetic regulation, fetal programming, overnutrition, undernutrition, micronutrients, human milk, microbiome, disease origin

## Abstract

Epigenetic modifications are among the most important mechanisms by which environmental factors can influence early cellular differentiation and create new phenotypic traits during pregnancy and within the neonatal period without altering the deoxyribonucleic acid sequence. A number of antenatal and postnatal factors, such as maternal and neonatal nutrition, pollutant exposure, and the composition of microbiota, contribute to the establishment of epigenetic changes that can not only modulate the individual adaptation to the environment but also have an influence on lifelong health and disease by modifying inflammatory molecular pathways and the immune response. Postnatal intestinal colonization, in turn determined by maternal flora, mode of delivery, early skin-to-skin contact and neonatal diet, leads to specific epigenetic signatures that can affect the barrier properties of gut mucosa and their protective role against later insults, thus potentially predisposing to the development of late-onset inflammatory diseases. The aim of this review is to outline the epigenetic mechanisms of programming and development acting within early-life stages and to examine in detail the role of maternal and neonatal nutrition, microbiota composition, and other environmental factors in determining epigenetic changes and their short- and long-term effects.

## Introduction

Following the so-called “Developmental Origins” hypothesis, introduced by Barker and Osmond 30 years ago (1) and based on early developmental plasticity, the environmental influence on health and disease has been progressively explored over the last decades.

Research on this issue has eventually led to the discovery of a “second genome,” which comprises human microbiome and, *via* its metabolites, actively interacts with the genome derived by sperm and egg, resulting in far-reaching epigenetic modifications (2, 3).

The term “epigenetics,” which literally means “on top of genetics,” defines a variety of processes that cause mitotically and meiotically heritable changes in gene expression without modifying the deoxyribonucleic acid (DNA) sequence; particularly, DNA methylation, histone modification, and non-coding RNA are the main mechanisms underlying epigenetic modifications.

The period of life during which epigenetic DNA imprinting activity is the most active lasts from conception to the second anniversary, thus being referred to as “the 1,000 days period” (4). During this time interval, *via* epigenetic changes, early nutrition can play a key role in developmental programming, thereby possibly influencing the individual susceptibility to the later development of cardiovascular diseases, obesity, diabetes, and other non-communicable chronic conditions.

According to recent evidence, the microbial colonization begins far before birth; particularly, the microbial flora from amniotic fluid, placenta, and maternal gut can support the development of a prenatal microbiota (5–7) that is likely to have an influence on the developing embryo and fetus. Furthermore, early postnatal microbiota perturbations, resulting from skin contacts, mode of delivery and neonatal diet, have been proposed to play a role in the susceptibility to several late-onset diseases (i.e., obesity, diabetes, allergies, asthma, autoimmunity) by modulating the immune development through epigenetic modifications (8). Similar mechanisms might also underlie the increased risk of necrotizing enterocolitis associated with the use of antibiotics or histamine-2 receptor blockers in the neonatal population (9, 10).

Recent evidence has shown that some of the epigenetic changes ensuing from early nutrition and microbiome can be transgenerationally inherited, thus having a significant impact on evolution (11). Although preliminary data currently available on the role of epigenetic in the determination of long-term health and disease look promising, however, most of the underlying mechanisms still need to be clearly elucidated.

**Figure 1:**
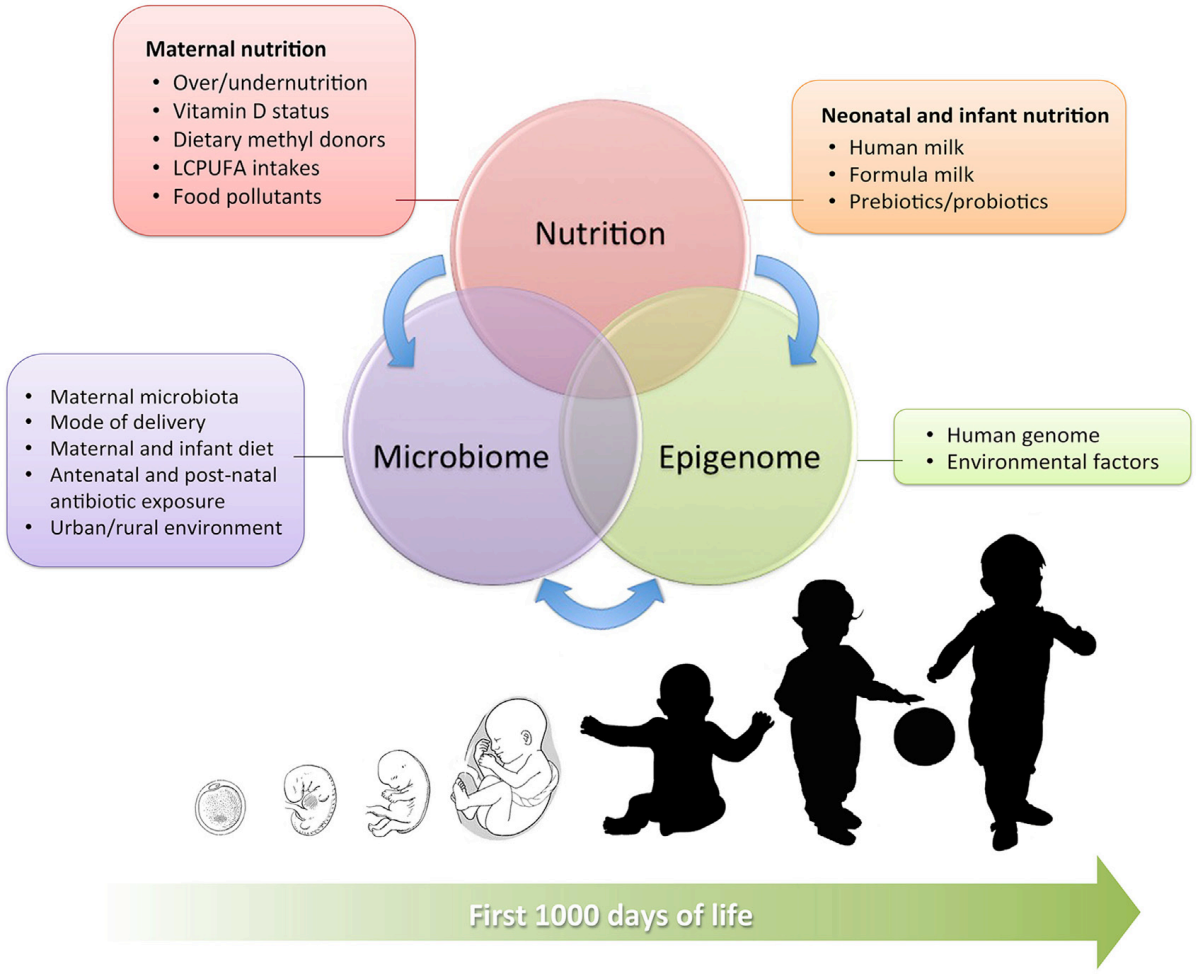
Interrelation between maternal and neonatal nutrition, gut microbiota, and epigenetics during the first 1,000 days of life. The main influencing factors are detailed in the boxes.

This review aims to provide a complete overview on the complex interactions between early nutrition, microbiome, and epigenome during the early phases of human development (summarized in Figure 1), examining current evidence in detail and shedding light on the complex epigenetic processes that have been identified so far.

## Effect of Maternal Nutrition on Pregnancy Epigenetics and Fetal Programming

Throughout their lifespan, humans are exposed to several environmental hazards; nevertheless, the effects of these exposures may take decades for their phenotypic expression. It has been speculated that, in response to maternal homeostasis and intrauterine stimuli, the fetus undergoes predictive responses theoretically resulting in permanent adjustments of the homeostatic systems, aimed at improving adaptation to the postnatal environment. Nevertheless, a mismatch may occur, and these adjustments might ultimately become disadvantageous, resulting in heritable risk factors for future diseases (12).

In this section, we will review the effects of maternal nutrition, in its qualitative [i.e., micronutrients, long-chain polyunsaturated fatty acids (LCPUFAs), food pollutants] and quantitative (i.e., under- and overnutrition) aspects, on epigenetic fetal programming in both human and animal models. A detailed list of these effects on the offspring is provided in Table 1.

**Table 1:** Main antenatal factors associated with epigenetic modifications in the offspring.

Factor	Epigenetic mechanism	Route	Clinical effects in the offspring
Maternal supplementation with dietary methyl donors (folic acid, vitamin B12, choline, zinc, methionine, betaine)	Deoxyribonucleic acid (DNA) methylation	Runt-related transcription factor 3 (Runx3)	Increased risk of allergic airway disease in offspring mice (16)

Maternal choline supplementation	DNA and histone methylation	Histone H3, Kmt1a, Kmt1c	Improved development and functioning of the adult rat brain (23)

Maternal zinc supplementation	DNA methylation (gut cells)	Not specified	Anti-inflammatory effects on the intestinal mucosa (26)

Maternal vitamin D deficiency	DNA methylation (placental tissue)	Vitamin D metabolic pathway (1α-hydroxylase, vitamin D receptor, retinoid X receptor)	Preeclampsia development in humans and possible adverse pregnancy outcomes (31)

Low maternal dietary intakes of long-chain polyunsaturated fatty acids	DNA methylation	Angiogenic factor genes	Vascular dysregulation, altered placentation, and increased long-term risk of cardiovascular diseases (32, 33)

Maternal high-fat diet	Histone acetylation	H3K9, H3K14, H3K18 in fetal liver	Alteration in fetal chromatin structure and fetal non-alcoholic fatty liver disease in primates (47)
	Histone acetylation	Hepatic antioxidant enzyme Pon1	Gender differences in the oxidative balance observed later on in life (49)
	Histone acetylation	Fetal surtuin 1 (SIRT1)	Increased susceptibility to fetal non-alcoholic fatty liver disease (50)

Maternal hyperglycemia	Histone modification	Insulin growth factor (IGF-1) promoter	Decreased hepatic IGF-1 mRNA variant levels and H3Me3K36 of IGF-1 gene in male rat offspring. Possibly increased susceptibility to adult-onset insulin resistance (38)

Maternal food restriction resulting in intrauterine growth restriction (IUGR)	DNA methylation	IGF-1 A and B genes; IGF-1 exon 1–2	Increased risk of obesity and related metabolic dysregulation in rats (39)

IUGR	Histone acetylation	Histone H3, peroxisome proliferator-activated receptor-γ coactivator 1 (PGC-1) and carnitine-palmitoyl-transferase I (CPTI) genes	Possibly increased susceptibility to insulin resistance and diabetes in rats (37)
	Histone methylation and acetylation	Pancreatic and duodenal homeobox factor-1 (PDX1) gene	Reduced PDX1 expression in rats; possible role on type 2 diabetes development (40)

Phthalates exposure	DNA methylation hypothesized	Adrenal and gonadal steroidogenesis pathways	Decreased circulating levels of testosterone and aldosterone in adult male offspring and of estradiol in adult female offspring in rats (58)
	Global DNA hypermethylation in CD4+ T cells	TH2 differentiation genes, including the GATA-3 repressor of zinc finger protein 1 (Zfpm1)	Increased risk for allergic airway disease (60)

Bisphenol A exposure	DNA methylation	Genes involved in mammary gland development	Increased mammary cancer risk in rats (63)

Maternal exposure to *Acinetobacter lwoffii* during pregnancy	Histone acetylation	T-helper 1 and T-helper 2 relevant genes in CD4+ T cells	Reduced risk of asthma-like disease in mice (156).

### The Role of Methyl Donors and Their Cofactors on Fetal Epigenetics

The most common epigenetic modification is DNA methylation, resulting from the addition of a methyl group to the cytosine of a cytosine–guanine pair. If this methylation is located in close proximity to a gene, it often results in lowered or abolished gene expression. As the one-carbon metabolism depends upon dietary methyl donors, DNA methylation can be influenced by nutrition during early life: this process involves a large number of enzymes with methyltransferase activity, cofactors including choline, methionine, vitamins B6 and B12, zinc, betaine and dietary micronutrients acting as methyl donors (13–15).

Folic acid is a well-known one-carbon donor for DNA methylation and synthesis; its role is crucial during early postnatal development, when rapid cell growth and proliferation take place. However, in animal models, *in utero* exposure to a maternal diet supplemented with methyl donors has also shown unexpected effects, such as an increased susceptibility to allergic airway disease. Particularly, mice born to mothers supplemented with folic acid, vitamin B12, methionine, zinc, betaine, and choline experienced significantly higher rates of allergic airway inflammation, ensuing from an excessive methylation of the runt-related transcription factor 3 (*Runx3*), a mediator of T-lymphocyte differentiation predisposing to asthma-like diseases (16). Hence, methylation can act as a double-edged sword, and this finding is consistent with human epidemiologic evidence of a significant association between perinatal folic acid supplementation and increased risk of wheezing at 18 months of age (17).

On the other hand, folic acid deficiency has been associated with an increased expression of inflammatory mediators, such as interleukin (IL)-β, IL-6, tumor necrosis factor-α (TNF-α), and monocyte chemoattractant protein-1 in the mouse monocyte cell line RAW264.7 (18); this is consistent with the beneficial role of folic acid in preventing inflammatory responses (19).

In addition to folic acid, imbalanced maternal concentrations of other micronutrients can affect DNA methylation patterns in the offspring. For example, increased maternal serum levels of vitamin B12 during pregnancy correlate with decreased global DNA methylation in newborns, while high vitamin B12 serum levels in newborns have been associated with reduced methylation of insulin-like growth factor-binding protein 3, a gene involved in intrauterine growth (20). Vitamin B12 deficiency, on the other hand, can result in global hypomethylation as, along with folic acid, this micronutrient is involved in the synthesis of methionine and S-adenosyl methionine, donors commonly required for the maintenance of DNA methylation patterns (21).

There is growing evidence that optimal dietary intakes of choline, which is involved in one-carbon transfer or methylation pathways as folic acid, support a successful completion of fetal development (22). In animal studies, maternal choline supplementation during pregnancy as been shown to modify histone and DNA methylation in fetal liver and brain, suggesting concerted epigenetic mechanisms that contribute to favorable long-term developmental effects (23).

Due to its role in DNA methylation, zinc status can exert a fundamental influence on the epigenome. Particularly, its deficiency during intrauterine life and childhood may contribute to alter promoter methylation, resulting in an immune dysregulation that could contribute to the development of chronic inflammatory diseases (24) and to increase cardiovascular risk (25). On the contrary, evidence from animal studies has shown that maternal zinc supplementation during pregnancy is associated with a lower degree of DNA methylation in gut cells, which, in turn, can have an anti-inflammatory effect on the intestinal mucosa (26).

In sum, these findings suggest that specific dietary interventions at key time points of fetal development can lead to different and unintended long-term consequences on health and disease.

### Vitamin D and Its Effect on Fetal Epigenetic Programming at the Placental Interface

Changes in vitamin D metabolism have been associated with altered methylation patterns in placental tissue, with a possible influence on pregnancy outcome and on the development of great obstetrical syndromes (27).

After oral ingestion, vitamin D3 undergoes hepatic and renal hydroxylation, being thus converted in its biologically active form (1,25-dihydroxyvitamin D3), which enters the cells and combines with the retinoid receptor, forming a heterodimer that binds to vitamin D-responsive genes and regulates their transcription and translation (28).

The placenta has a functional vitamin D endocrine system, expressing vitamin D receptors and allowing the local conversion of vitamin D in its active form (29). Of interest, a potential association between vitamin D insufficiency and increased risk of preeclampsia has been reported (27, 30). In order to better understand the possibly underlying epigenetic processes, Anderson et al. (31) analyzed DNA methylation patterns and protein expression of placental genes involved in vitamin D metabolism in relation to vitamin D intakes in women with preeclampsia, compared to those who remained normotensive throughout pregnancy. Though not significant, the incidence of vitamin D deficiency was higher in the preeclamptic (46%) than in the normotensive group (20%). Moreover, placental samples from pregnant women with preeclampsia showed increased DNA methylation of *CYP27B1*, vitamin D receptor and retinoid receptor genes, with lower protein expression levels of the latter. A possible interference between availability of vitamin D at the maternal–fetal interface, hypermethylation of key placental genes involved in vitamin D metabolism and placentation can be hypothesized; however, further studies are needed to clarify the exact mechanisms.

### LCPUFAs Intakes and DNA Methylation

Long-chain polyunsaturated fatty acids are essential components of human diet known for their beneficial effects on health, growth and development. From an epigenetic point of view, LCPUFAs are rich in phospholipids, which are among the major methyl group acceptors in the one-carbon metabolic pathway, being thus involved in methylation reactions (32).

Recent studies investigating the role of LCPUFAs in determining gestational outcomes and influencing the offspring’s health have shown that inadequate intakes during pregnancy may result in aberrant DNA methylation patterns, affecting the expression of clinically relevant genes (e.g., angiogenic factor genes) (32). This can contribute not only to the vascular dysregulation associated with abnormal placentation but might also play detrimental effects on fetal programming, ensuing in an increased long-term risk of cardiovascular diseases (33).

High intakes of n-3 LCPUFAs, such as eicosapentaenoic acid, docosahexaenoic acid (DHA) and α-linolenic acid (18:3n23) are largely known to be associated with protective metabolic effects. In mice, high-dietary n-3 LCPUFAs have been shown to bring significant epigenetic changes in leptin promoter, resulting either from the inhibition of the enzymes catalyzing DNA methylation and histone modifications or from a reduced availability of the relative substrates (34). On the other hand, fish oil supplementation in animal models has been associated with decreased global DNA methylation in the liver persisting for two generations, lowered blood lipid concentrations, increased insulin-stimulated glucose uptake, and insulin sensitivity (35); however, the pathways involved still need to be completely elucidated.

### Epigenetic Effects of Maternal Undernutrition and Overnutrition during Pregnancy

Growth and development during fetal life and early childhood are greatly influenced by macronutrient intakes during pregnancy, suggesting the importance of an adequate maternal diet in this crucial phase. A poor maternal nutrition has been shown to modify epigenetic programming and have a negative impact on fetal gene expression, resulting in possible long-term consequences (36).

Maternal undernutrition (either global or protein-restricted) has been extensively used in rodent studies to induce intrauterine growth restriction (IUGR). In the setting of an altered early nutrition, organisms can achieve environmental adaptation by modulating their gene expression *via* epigenetic alterations of histone markers. In experimental models of IUGR, uteroplacental insufficiency has been shown to decrease postnatal insulin-like growth factor-1 (*IGF1*) mRNA variants and H3 acetylation of *IGF1* gene (37). Similar patterns of histone modification on *IGF-1* promoter have also been reported by Zinkhan et al. (38) following maternal gestational hyperglycemia. Moreover, different changes in hepatic IGF-1 mRNA expression and histone H3K4 methylation have also been reported in relation to early growth patterns following IUGR in the rat (39). One of the molecular phenotypes associated with IUGR rats is a decreased expression of pancreatic and duodenal homeobox factor-1 (*PDX1*), a key transcription factor regulating pancreatic development (40); similar findings have also been observed for the muscular glucose transporter GLUT4 (41).

Low-protein regimens at conception or during pregnancy bring additional evidence on the noxious epigenetics effects of maternal nutrition deficiency on the offspring health: in animal models, a protein-restricted maternal diet has been associated with impaired immune response (42), increased sensitivity to oxidative stress (43), metabolic abnormalities, and glucose dyshomeostasis (44).

The Dutch Winter Study provides a bright example on how famine during early and mid-gestation can lead to metabolic dysregulation later on in life, having been associated with hyperglycemia, higher incidence of coronary heart disease, a more atherogenic lipid profile, disturbed blood coagulation, increased stress responsiveness, and obesity (45). Remarkably, follow-up studies on the population exposed to the Dutch famine have shown increased rates of neonatal adiposity and poorer health among the offspring of women exposed to maternal undernutrition *in utero*, suggesting that the related detrimental effects are probably transmitted to subsequent generations through epigenetic modifications persisting during meiosis (46).

In addition to nutrient deficiency, overnutrition during pregnancy has also been demonstrated to cause detrimental effects on the offspring health either at early or late life stages.

Studies performed in primates have shown that a high-fat maternal diet can alter fetal chromatin structure *via* covalent histone modifications (47). Maternal high-fat dietary intakes have been associated with hormonal dysregulation and release of inflammatory cytokines that can predispose the offspring to various vascular diseases (48). Strakovsky et al. (49) showed that, by modifying histone acetylation, a maternal diet with high-fat contents contributes to alter the expression of neonatal hepatic antioxidant enzymes in a sex-specific manner, thus possibly contributing to the known gender differences in the oxidative balance observed later on in life. In primates, a maternal high-fat diet has been reported to modulate protein deacetylase activity and to modify fetal surtuin 1 (*SIRT1*) histone, which is a likely mediator of fetal epigenome and metabolome in the setting of maternal obesity (50).

As for maternal obesity, in further animal models it has been correlated with increased pancreatic beta cell mass and excessive insulin secretion (51) that, in the long-term, can predispose to the development of diabetes and non-alcoholic fatty pancreas disease in the offspring (52).

Eventually, of great interest is the effect of nutrition on microRNAs (miRNAs), which reciprocally interact with other epigenetic mechanisms, such as histone modification and DNA methylation, in order to modulate the expression of target genes (53). The expression of miRNAs can be controlled by DNA methylation and chromatin modifications; in turn, miRNAs affect the methylation machinery and the expression of proteins involved in histone modification. The combination of these mechanisms contributes to determine gene expression and the resultant phenotype.

Nutrition has been shown to modulate the expression of endogenous miRNAs, resulting in different serum miRNA profiles that may influence biological processes, including inflammation and metabolism. Overnutrition can actively regulate several miRNAs involved in immune function modulation, thus contributing to the development of chronic inflammation (54). Of interest, a maternal high-saturated-fat diet has been shown to induce inflammation pathways in the offspring in animal (55) and human studies (56).

Eventually, the intrauterine exposure to inflammatory mediators, whose levels are often increased in the context of obesity and metabolic dysregulation, has been shown to influence the adulthood risk of diseases with an inflammatory component (e.g., asthma, cardiovascular diseases, atherosclerosis) (48, 57). Although the causative mechanisms still need to be elucidated, a detrimental effect of maternal inflammation on the appropriate maintenance of fetal epigenetic profiles can be hypothesized.

### Epigenetic Effects of Antenatal Exposure to Food Pollutants

The epigenetic effects of prenatal exposure to chemical food contaminants, such as phthalates and bisphenol A (BPA), are in the early stages of elucidation.

Phthalates are ubiquitous plasticizers mainly used in the manufacture of polyvinyl chloride products; food packaging and contact materials are considered the major sources of food contamination. Phthalates have been shown to act as endocrine-disrupting compounds and are thought to interfere with epigenetic programming. In a rat model, fetal and postnatal contamination through maternal exposure and food sources, respectively, has been found to decrease circulating levels of testosterone and aldosterone in adult male offspring and of estradiol in females (58). Furthermore, recent murine evidence has contributed to shed light on the epigenetic mechanisms undergoing the increased risk for allergic airway disease observed after prenatal and early postnatal phthalate exposure (59), showing a significantly increased global DNA hypermethylation in CD4+ T cells of the offspring that resulted in a transcriptional downregulation of genes involved in T-helper 2 differentiation (60).

Contamination modalities for BPA are similar to those previously described for phthalates. Dietary exposure to BPA in the Agouti viable yellow (A(vy)) mouse model has been shown to hypomethylate metastable epialleles, an effect that can be counteracted with dietary supplementation of methyl donors or genistein (61). According to further evidence from animal models, antenatal and perinatal BPA exposure has been found to lead to specific epigenetic changes, resulting, among the others, in impaired stress response (62) and higher rates of mammary cancer (63); this contributes to highlight the clinical burden associated with chemical food contamination.

## Early Nutrition and Epigenetics: The Key Role of Human Milk

Among the postnatal factors that can contribute to determine lifelong health and disease *via* epigenetic mechanisms, infant feeding plays a key role. Evidence on the epigenetic effects of early nutrition on developmental programming, possibly resulting in later development of cardiovascular diseases, overweight, obesity, diabetes, and other chronic conditions (64), is constantly increasing.

Human breast milk (HBM) is universally considered the normative standard for infant feeding, as it confers unique nutritional and non-nutritional benefits that could be partly explained by epigenetics; although the majority of the underlying mechanisms are still unclear, a number of them, summarized in Table 2, have been progressively elucidated.

**Table 2:** Main epigenetic modifications associated with neonatal nutrition.

Type of feeding	Epigenetic mechanism	Route	Clinical effects
Human breast milk	Unclear	Nuclear factor-κB (NF-κB) pathway inhibition	Decreased secretion of interleukin (IL) 8 in human intestinal cells; possible protective effect on necrotizing enterocolitis (NEC) development (65)
	Unclear	Peroxisome proliferator-activated receptor-γ (PPARγ)	Counterbalance of the increased risk of obesity associated with PPARγ2 Pro12Ala polymorphism in adolescents (71)
	Unclear	Hepatic hydroxymethyl glutaryl coenzyme A reductase	Lower serum levels of total cholesterol and low-density lipoprotein cholesterol in adults who were breastfed as infants (73)

Formula feeding	Histone hyperacetylation	Inflammatory and pattern-recognition receptor genes (including IL-8 and toll-like receptor 4)	Mild lesions on intestinal mucosa; possible predisposing role for NEC development (77)

Lactoferrin is an abundant HBM protein that regulates gene expression by binding to pro-inflammatory bacterial DNA sequences in Peyer’s patches and intestinal mucosa. As a result, this binding inhibits the transcriptional activation of nuclear factor-κB (NF-κB) in human intestinal B-lymphocytes and downregulates IL-8 synthesis, which is involved in the pathogenesis of necrotizing enterocolitis (65).

Human milk oligosaccharides contained in HBM promote a healthier composition of gut microbiota, which plays a leading role in programming the infant’s immune phenotype and in preventing early and later diseases (66). Particularly, breastfeeding-induced microbiota has been proved to regulate the expression of genes involved in digestion, barrier function, and angiogenesis and enhance immunoglobulin-A secretion (67), thus possibly contributing to prevent necrotizing enterocolitis. Similar mechanisms involving gut microbiota have also been proposed to explain HBM beneficial effects in preventing infections and immune-mediated diseases, such as asthma and allergies (68).

The most striking evidence of nutritional programming, however, is observed for the protective effect of HBM on later obesity and metabolic diseases. The peroxisome proliferator-activated receptor-γ (*PPARγ*) transcription factor is highly expressed in adipocytes, where it regulates the maintenance of insulin sensitivity. In particular, *PPAR*γ ligands contribute to restore normal levels of adipose-derived substances, such as leptin, adiponectin, and TNF-α, thus reversing insulin resistance syndrome, improving endothelial cell functions and reducing inflammation (69). Pro12Ala substitution at codon 12 is the most common variant identified in the *PPAR*γ*2* gene and has been associated with an increased risk of obesity in adulthood (70). Non-breastfed adolescents carrying Pro12Ala polymorphism showed higher body mass index, waist circumference, and higher skinfold thickness when compared to those who had been breastfed even for a short period. According to this finding, by inducing epigenetic modifications, breastfeeding could counterbalance the risk of obesity even in genetically predisposed adolescents (71). To this regard, the authors supposed a possible role of the natural *PPARγ* ligand contained in HBM in decreasing *PPARγ2* transcriptional activity in Pro12ala carriers.

Human breast milk is particularly rich in n-3 LCPUFAs, such as DHA. In animal models, DHA has been shown to downregulate hepatic lipogenesis and cholesterol biosynthesis pathways (72), consistently with the lower levels of serum total cholesterol (TC) and of low-density lipoproteins (LDL) observed in adults who had been breastfed as infants (73). Paradoxically, however, increased TC and LDL levels have been observed in breastfed infants during their first year of life (73). This effect, which is rapidly reversible, is likely to be a direct consequence of the high cholesterol contents of HBM that, on the other hand, contribute to reduce TC and LDL levels at older ages by downregulating hepatic hydroxymethyl glutaryl coenzyme A reductase *via* epigenetic modifications.

The beneficial effects of HBM are not only limited to breastfed infants but are also relevant to breastfeeding mothers. An inverse correlation between breastfeeding duration and breast cancer risk has been previously established (74). This effect has also been observed in women carrying deleterious mutations in the *BRCA1* gene who breastfed their children for more than 1 year (75). Although the epigenetic links occurring between HBM components and breast cancer prevention need to be further elucidated, a proposed underlying is the inhibiting effect that DHA exerts on breast cancer cell growth by modulating *PPAR*β mRNA expression (76).

While the beneficial epigenetic effects of HBM are well established, on the other hand, less is currently known about formula feeding.

Recent evidence from preterm piglets has shown that, compared to colostrum, formula feeding was associated with a significant upregulation of inflammatory and pattern-recognition receptor genes, including IL-8 and toll-like receptor (TLR)-4; this pro-inflammatory status corresponded to decompacted chromatin configurations and histone hyperacetylation in key inflammatory genes, thus suggesting underlying epigenetic modifications. These findings histologically correlated with mild mucosal lesions, and a possible predisposing role for NEC development has been hypothesized (77).

## Intestinal Microbial Colonization and Epigenetics

The gastrointestinal tract is the most important site of host–microbe interactions and the establishment of an indigenous intestinal microbiota during early life has been shown to have a major impact on human physiology.

Compelling evidence contradicts the dogma that the fetus resides in a sterile environment and that the newborn only attains its microbiota after extrauterine exposure (78). Although controversy still exists about the “sterile womb” versus the “*in utero* colonization” concept (79), it is difficult to dispute that the microbial environment of the fetus *in utero* has major implications for health and disease. For over 30 years, we have known that even without a ruptured amniotic sac, amniotic fluid frequently contains significant levels of bacteria as evaluated by quantitative culture techniques (80). Moreover, intrauterine “infection” and subsequent inflammatory responses link to prematurity, brain, lung, and eye disease (81, 82) and suggest that perturbations of critical maternal–fetal–microbial interactions lead to pathology.

The postnatal establishment of gut microbiota is influenced by several factors: mode of delivery, contact with the mother (such as with skin-to-skin care), composition of the diet, and administration of pharmacologic agents, especially antibiotics (83). Immediately after birth, the infant gut microbiome has relatively low-species diversity and high rates of bacterial flux. By 3 years of age, this flux begins to stabilize (84). *Staphylococcus, Streptococcus, Escherichia coli*, and *Enterobacteria* are thought to be the first colonizers of the gut. Facultative anaerobic bacteria subsequently replace these taxa and consist in large relative abundances of Actinobacteria and Firmicutes (85). This is influenced in large part by diet; for example, breastfeeding appears to stimulate the growth of *Bifidobacteria* species (86).

Hence, early environmental influences influencing gut microbiota during this crucial developmental period can modify its composition toward more pathogenic profiles that, in turn, can persist until adulthood and exert long-lasting effects on health and disease.

Among the possible mechanisms through which intestinal bacteria can influence human health, epigenetic modifications prevail. The direct potential of microbes to induce epigenetic changes in the host has been recently demonstrated by the evidence of microbe-specific patterns of epigenetic DNA modification after exposure to commensal or pathogenic organisms in immature human cells from intestinal epithelia (87). Interestingly, the same study also reported that prenatal glucocorticoid-induced epigenetic programming results in an altered gut microbiota composition in mice, suggesting the existence of complex interactions between microbiome and epigenome (87).

The role of microbiome as an epigenetic modulator is thus gaining increasing attention and, although the underlying mechanisms still need to be partially elucidated, current evidence supports a significant correlation between gut microbiome composition and epigenetic changes in genes relevant to immunological, metabolic, and neurological development and functions. The role of gut microbiota on each of these areas is addressed in the following paragraphs; moreover, a summary of the currently recognized epigenetic mechanisms associated with specific gut microbiota profiles is provided in Table 3.

**Table 3:** Epigenetic modifications associated with specific profiles of gut microbiota.

Strains	Epigenetic mechanism	Route	Clinical effects
*Lactobacilli* and *Bifidobacteria*	Butyrate-associated histone deacetylase (HDAC) inhibition	Nuclear factor-κB, peroxisome proliferator-activatedreceptor-γ, interferon-γ	Reduced intestinal and systemic inflammation (88, 104)
	Deoxyribonucleic acid (DNA) methylation secondary to methyl-donor production	Genes involved in inflammatory pathways	Modulation of intestinal and systemic inflammation (88)

Increased Firmicutes/Bacteroidetes ratio	DNA methylation (CpG)	Toll-like receptor (TLR) 2 and TLR-4	Altered expression of pro-inflammatory genes
			Increased risk of type 2 diabetes mellitus (118)
	DNA methylation	SCD5 gene, encoding for a primate-specific stearoyl- coenzyme A desaturase	Altered catalysis of monounsaturated fatty acids from saturated fatty acids
		USF gene, involved in fatty acid synthase and in lipogenesis	Possibly increased risk of overweight, obesity and lipid metabolism disturbances (119)

### Modulation of Immune Response and Development of Immune-Mediated Diseases

Gut microbiota plays a key role in the development of immune response since the early phases of life: by activating specific pathways of molecular signaling, it supports the maturation of gut-associated lymphoid tissue (88), promotes the conversion of CD4+ T-cells into T-regulatory cells (89), and influences the balance between T-helper 1 and 2, which is known to have significant effects on the development of allergic diseases (90). Furthermore, specific microbiota-induced patterns of TLR-2 and TLR-4 expression have been observed in gut cells; by modulating TLR expression, commensal intestinal bacteria may prime the host response to pathogenic threats and act as local immunomodulators, suppressing pro-inflammatory pathways and promoting the intestinal transcription of cytoprotective genes (91).

Intriguing data from experimental animal models have supported the existence of a causal relationship between early microbial contact and the development of immune system: particularly, mice reared in germ-free conditions failed to develop immune tolerance and were more prone to allergic-type immune responses (92).

Over the last decade, increasing evidence has consistently shown that an abnormal composition of gut microbiota in the early phases of life is associated with the subsequent development of immune-mediated diseases (93, 94). The intrauterine period, as well as post-partum, is crucial for the establishment of immune response; therefore, different profiles of intestinal bacteria in this delicate phase can result in different long-term effects on immunological functions.

During vaginal delivery, infants receive a significant inoculum of colonizing microbes from maternal birth canal and intestine. Consequently, neonates born by C-section (CS) delivery exhibit aberrant gut colonization patterns (95), which may extend at least until the age of 7 years (96). According to an epidemiological study on 1.9 million subjects from Denmark (97), the incidence of immune-mediated disease including asthma, juvenile arthritis and inflammatory bowel disease (IBD) is significantly higher in CS-born children.

In a similar fashion, treatment with antibiotics is known to cause drastic changes in intestinal microecology and, although other potential confounding factors may be at play (98), early antibiotic exposure in human and animal studies has been associated with increased long-term risk of asthma (99), type 1 diabetes (T1D) (100), and IBD (101). However, the effects of microbiota composition in predisposing to non-communicable diseases are more likely to ensue from the concerted interactions occurring among intestinal bacterial profiles, their metabolites, and the host’s responsiveness rather than from the effects of single bacterial strains.

Recent data from pediatric patients with both ulcerative colitis and Crohn’s disease has shown a striking reduction in species richness and diversity in their microbiota, with particularly low abundance of *Lactobacilli, Bifidobacteria*, and other bacteria that are known to have a positive influence on gut homeostasis, such as *Eubacterium rectale* and *Faecalibacterium prausnitzii* (102). Given the substantial stability of intestinal microbiota after the first years of life (84), it is likely that these abnormal patterns may establish in earlier phases, contributing to predispose toward IBD development by inducing epigenetic changes. As an example, *Lactobacilli* and *Bifidobacteria*, whose concentration is lowered in children with IBD, exert well-known anti-inflammatory effects and support the integrity of intestinal barrier by producing butyrate through cross-feeding (103). Acting as a histone deacetylase (HDAC) inhibitor, butyrate can dampen gut inflammation by suppressing nuclear factor-B (NF-B) activation (88), upregulating the expression of *PPAR*γ and decreasing interferon-γ production (104). In addition, *Lactobacilli* and *Bifidobacteria* can also affect DNA methylation by regulating methyl-donor availability through their production of folate (105). Hence, a decreased butyrate production and folate bioavailability ensuing from an altered microbiota may result in an increased expression of inflammatory pathways that, in turn, can predispose to intestinal and systemic inflammation (88).

Further evidence on how early modifications of gut microbiota can influence immune responses is provided by the non-obese diabetic mouse model of human T1D. Dysfunctional antigen-presenting cells (APCs), ensuing in aberrant immune tolerance, have been associated with T1D development (106). Recently, it has been shown that prenatal exposure to different antibiotics, altering gut bacterial composition at the earliest phases of life, is associated with significant differences in the autoantigen-presenting functions of APCs, resulting in either protective (107, 108) or more diabetogenic immune profiles (100). Of interest, the observed protective effects were heritable by the second-generation offspring and were also transmitted to other hosts *via* gut microbiota transfer, suggesting the presence of underlying epigenetic mechanisms (108).

### Long-term Metabolic Effects of Early Aberrant Microbiota

The association between early antenatal and perinatal factors and metabolic profile later in life has been largely established. As an example, preterm birth has been extensively associated with the development of obesity, cardiovascular diseases, and type 2 diabetes (T2D) in adulthood (109, 110). A significant correlation among maternal and neonatal nutrition (73), antenatal (111) and postnatal antibiotic exposure (98), and later metabolic outcomes is also well-known, and a recent meta-analysis of epidemiological studies has shown that the risk of childhood obesity is significantly increased in infants born by CS, even after correcting for maternal weight (112).

Similar metabolic effects have been associated with early aberrant profiles of gut microbiota (113–115), thus suggesting that the impact of microbial contact on metabolic maturation is most profound during early life and healthy metabolic development depends on interactions with healthy microbiota. Of interest, the obese phenotype induced by early aberrant gut microbiota has been observed to persist even after gut microecology is restored (116), and it has also been shown that obesity may be transferred to germ-free mice by colonizing them with intestinal microbes from obese humans (117).

The role for microbiota-induced epigenetic modifications in mediating these effects is increasingly emerging; nevertheless, the mechanisms underlying the interactions between early-life microbiota and epigenetic programming of host metabolic physiology are only beginning to be unraveled.

According to Remely et al. (118), obese adults or subjects with T2D exhibited marked differences in gut microbiota composition compared to lean individuals. The metabolic state of the host correlated also with innate immune function, as significantly increased CpG methylation was discovered in the regulatory region of *TLR4* gene in obese subjects compared to lean individuals; moreover, the promoter region of the *TLR2* gene showed higher methylation levels in the diabetic group compared to healthy controls.

Deciphering the direction of causality between gut microbiota composition and the epigenetic modulation of microbial defense is difficult. Further insights into the sequence of events are provided in a report by Kumar et al. (119), according to which distinct DNA methylation profiles were detected in blood samples obtained from women 6 months after delivery, depending on the predominance of either Firmicutes or Bacteroidetes and Proteobacteria in the fecal microbiota during pregnancy. The potential clinical significance of this finding is highlighted by previous evidence indicating that the ratio of intestinal Bacteroidetes to Firmicutes is associated with metabolic disorders, and by the observation that the differences in methylation were located in genes whose function is linked to obesity, metabolism, and inflammation.

### The Microbiota–Gut–Brain Axis

The first 1,000 days of life are crucial not only for the physiologic establishment of gut microbiota, but also for the development of central nervous system (CNS). While the reciprocal interaction between the brain and intestinal organs has been recognized long ago (120), evidence supporting a correlation between microbiota composition and altered neurocognitive and behavioral development has progressively emerged in the last decade, contributing to outlining the so-called “microbiota–gut–brain axis” (121).

Remarkable evidence on the effects of gut colonization on brain functions and development is provided by rodents raised in a sterile environment, which thus lack gut microbiota and are referred to as germ free (GF) (122). The most commonly reported phenotype in GF mice was an increased anxiety-related behavior (123). This altered behavioral response was accompanied by changes in the concentrations of neurotransmitters, their metabolites and neurotrophic factors involved in neural plasticity (124). Although the underlying molecular mechanisms are not well-understood, microbiota-related epigenetic regulation of gene expression and transcription in different brain regions has been hypothesized (125). Furthermore, increased basal levels of corticosterone and enhanced responses to stressors, which are known to negatively influence brain development (126), have also observed in GF animals, thus suggesting a possible role for gut microbiome in the regulation of hypothalamic–pituitary–adrenal axis (127).

Of interest, the reconstitution of normal microbiota patterns early in life in GF mice normalized both behavioral patterns and neurotransmission concentrations (125), whereas such an effect could not be established in adulthood (128, 129). Besides suggesting the role of microbial colonization in initiating signaling mechanisms that affect neuronal circuits involved in brain development and behavior (125), these results also suggest the existence of a critical window for intestinal microbes to influence developmental programming of long-lasting brain functions. In a similar fashion, GF mice have also shown deficits in social functioning, completely reversible after post-weaning microbial gut colonization (130).

Further supportive evidence on the role of gut microbiome in modulating brain development and behavior is provided by the evidence that specific microbial profiles resulting from infections, antibiotic treatment, or administration of probiotic bacteria have been associated with consistent behavioral changes in rodent studies. As for the bacterial strains possibly involved, preliminary evidence supports *Bifidobacteria* and *Lactobacilli* in exerting positive behavioral effects (131, 132).

Among the pathways through which gut microbiome may modulate CNS functions and development, which are just beginning to be unraveled, a possible role for immune signaling has been proposed (133). As previously discussed, the multiple antigenic stimuli provided by early gut colonization are fundamental for an appropriate immunological maturation and have been proved to modulate the expression of immune-related genes *via* epigenetic changes. In turn, cytokine receptors have been revealed on neurons and glial cells (134) and a significant contribution of immune signaling in normal brain function as well as during aging or in the context of neurodegenerative diseases (135–138) has been observed, thus contributing to support this hypothesis.

Recently, a possible correlation between autism-spectrum disorders (ASD) and abnormal gut microbiota composition and metabolism has been hypothesized, basing on observation that gastrointestinal symptoms are a common comorbidity in ASD children (139). However, current evidence from human (140–142) and rodent (130) studies is limited and controversial, and large prospective and randomized trials are needed to shed further light on this issue.

The most striking example supporting the epigenetics role in the context of microbiota–gut–brain axis is provided by irritable bowel syndrome (IBS). Increased visceral sensitivity, ensuing from abnormal brain responses to physiological visceral stimulation, has been widely proposed as one of the key mechanisms underlying IBS clinical manifestations (143). In order to investigate the possible influence of epigenetics in determining brain susceptibility to visceral stimuli, Tran et al. exposed mice from a rodent IBS-like model to cerebral injections of trichostatin A (TCA), a potent HDAC inhibitor (144). According with their results, TCA injections led to a significant improvement in visceral hypersensitivity, quantified by the number of gut contractions in response to graded colorectal distension, thus supporting a possible involvement of epigenetic mechanisms in modulating stress-induced visceral pain and hinting a potentially beneficial effect of HDAC inhibition in the treatment of IBS. In line with this, altered microbiota profiles, whose association with detrimental epigenetic changes has been previously discussed, are a common finding in both pediatric and adult IBS patients (145, 146) and therapeutic trials with specific probiotic strains, such as butyrate-producing *Bifidobacteria*, have been shown to ameliorate visceral hypersensitivity in animal (147) and human studies (148). However, further studies are needed to elucidate epigenetic modifications of genes relevant to visceral pain in relation to gut microbiome (123) and its influencers during the early phases of life.

In sum, according to the abovementioned evidence, a thorough understanding of how the microbiome–gut–brain axis operates during infancy may provide not only useful insights into early neurocognitive development with possible translational applications but also a greater awareness on the several modifiable factors influencing the establishment of infant microbiome during the first 1,000 days of life and their crucial long-term effects (149).

### Therapeutic and Preventive Implications of Early Interventions on Gut Microbiota

Evidence on epigenetic programming by gut microbiota may be interpreted to form a basis for a hypothesis according to which detrimental epigenetic modifications and, consequently, the development of disease, might be prevented by modulating microbial contact in early life. Modifying gut microbiota through prebiotic, probiotic, and symbiotic administration [the so-called “bacteriotherapy” (150)] might represent a promising approach to rebalance the homeostasis of systemic and mucosal immune systems.

So far, the most extensive evidence of early microbial interventions has been published in the case of reducing the risk of atopic disease using probiotics in high-risk populations. Maternal and infant probiotic supplementation from the last weeks of pregnancy until the offspring was 6 months of age has been reported to significantly reduce the incidence of atopic dermatitis (151). Interestingly, this protective effect was still detectable at the age of 7 years (152), thus suggesting the existence of immune programming mechanisms, even though still largely unknown. Another clinical trial demonstrated that a solely maternal probiotic intervention during pregnancy and breastfeeding can effectively reduce the incidence of atopic dermatitis in the child (153), and a recent meta-analysis of clinical trials (154) concluded that, in order to be effective in decreasing the risk of atopic dermatitis, the probiotic intervention should be commenced before birth.

Inflammatory bowel disease and T1D are other examples of immune-mediated diseases that would possibly benefit from early probiotic interventions. As different bacteria can induce different immune responses, gut microbiota would represent an optimal target for preventive and therapeutic strategies aimed, for example, at generating self-tolerogenic APCs with protective effects toward T1D development (107), or at hindering the establishment of bacterial profiles that are known to upregulate pro-inflammatory pathways and predispose to IBD development (88).

Epigenetic programming is known to take place during fetal life, and microbial contact might have an impact on disease risk even before birth. This notion is consistent with observations according to which children whose mothers have lived in a farming environment during pregnancy, being thus more exposed to microbial antigens as compared to urban mothers, display a lower risk for asthma (155). This protective effect may be mediated by epigenetic mechanisms, since gestational exposure to *Acinetobacter lwoffii F78*, a microbe isolated from cowsheds, resulted in modulation of histone acetylation of key immune mediators and protected the offspring from the development of asthma-like disease in an experimental mouse model (156).

The novel evidence suggesting that microbial gut colonization may begin *in utero* (80) open new areas of research aiming both at understanding microbial epigenetic programming during fetal life and at devising maternal interventions to modify disease risk in the offspring, with potentially useful clinical implications.

## Conclusion

Evidence on the role of maternal diet, early nutrition, and gut microbiota in the establishment of lifelong health and disease by determining epigenetic modifications that can be transgenerationally inherited has progressively spread over the last decades.

In addition to a qualitative assessment, aimed at identifying the molecular pathways and the bacterial patterns of gut colonization involved, a quantitative evaluation would help to establish the threshold levels of exposure to nutrients deficiencies or to other noxious environmental factors that can lead to clinically relevant epigenetic changes. Moreover, a better understanding of the underlying mechanisms may be fundamental to improve the approach to disease prevention. Nevertheless, further experimental research linking together technology advances and bioinformatics analyses is needed to identify new key markers for translational studies of disease prediction and treatment in the human population.

## Author Contributions

FI, SM, RF, LC, FC, GL, SAM, JN, SR, GRS, and FR contributed to draft the paper, critically reviewed its content, and approved the final version submitted for publication.

## Conflict of Interest Statement

LC, FC, GL, SM, SAM, SR, GS, and FR have no conflict of interest to declare; FI has participated as a clinical investigator and/or consultant and/or speaker for Arla Food, Biogaia, Noos, Nestlè, and Nestlè Nutrition Institute, Wyeth, Danone. RF serves as a speaker for Biogaia and Plasmon Italia; JN is a Consultant for Infant Microbial Therapeutics and is in Scientific Advisory Board of Medela.

## Abbreviations

APC, antigen-presenting cell; ASD, autism-spectrum disorders; BPA, bisphenol A; CNS, central nervous system; CS, C-section; DHA: docosahexaenoic acid; DNA, deoxyribonucleic acid; GF, germ free; HBM, human breast milk; HDAC, histone deacetylase; IBD, intestinal bowel diseases; IBS, irritable bowel syndrome; IL, interleukin; IUGR, intrauterine growth restriction; LCPUFAs, long-chain polyunsaturated fatty acids; LDL, low-density lipoproteins; miRNAs, microRNAs; TC, total cholesterol; TCA, trichostatin A; TLR, toll-like receptor.
